# Editorial: The Psychology of Magic and the Magic of Psychology

**DOI:** 10.3389/fpsyg.2016.01358

**Published:** 2016-09-16

**Authors:** Gustav Kuhn, Jay A. Olson, Amir Raz

**Affiliations:** ^1^Department of Psychology, Goldsmiths, University of LondonLondon, UK; ^2^Cognitive Neuroscience Laboratory, Psychiatry Department, McGill UniversityMontreal, QC, Canada

**Keywords:** magic, science of magic

## Background

Conjurors are masters of illusion and deception, and they have developed astonishing methods for manipulating our experience. Intuitively, the link between magic and psychology seems obvious: magicians use techniques such as misdirection to manipulate our attention, illusions to distort our perception, and forcing to influence our decisions. Some of the early pioneers in Psychology (e.g., Binet, 1894; Triplett, 1900) recognized this close link between magic and psychology and published fascinating scientific papers investigating conjuring techniques. Although some researchers have used magic tricks to study cognition indirectly (e.g., developmental psychologists), few have attempted to bind magic to the science of psychology.

In 2005, Kuhn and Tatler published one of the first recent papers on misdirection, which illustrated how conjuring principles can be used to study visual attention (Kuhn and Tatler, 2005). Whilst this paper attracted much popular interest, many scientists at the time were skeptical about the idea of using magic to explore the inner working of the mind. Although the relationship between magic and psychology is intuitive, this approach requires new paradigms and possibly new ways of thinking about cognitive mechanisms. However, because few researchers have access to the secret armamentarium of magical techniques, studying magic scientifically became the privilege of a small group of investigators with direct experience in conjuring. And yet, the last decade has seen a surge in research papers that have used magic to explore a wide range of topics in psychology. Concrete frameworks now explain how magic can be studied scientifically and the advantages that this direction may provide (Kuhn et al., 2008; Macknik et al., 2008; Demacheva et al., 2012). What was once a field restricted to a few scientists has rapidly grown into a vibrant research domain.

Whilst much of the research has focused on misdirection (for review see Kuhn and Martinez, 2012), the psychology of magic has expanded into fields such as decision making (Olson et al., 2015), problem solving (Danek et al., 2014), object permanence (Beth and Ekroll, 2014), pattern completion (Barnhart, 2010; Ekroll et al., 2013), belief formation (Parris et al., 2009; Subbotsky, 2010), visualmotor action (Cavina-Pratesi et al., 2011), sense of agency (Olson et al., 2016), and perceptual anticipation (Kuhn and Land, 2006; Kuhn and Rensink, 2016).

Inspired by the number of magic-related articles published in recent years—as well as the group of young researchers working in the field—we hoped to bring together different approaches that have used magic to investigate the mind. We had three main motivations for this research topic:

1. Collect a broad range of empirical papers that use magic to explore areas of cognition.
2. Help bridge the gap between magic theory and scientific theories of cognition.
3. Explore ways in which science could improve magic.

While most the papers in this issue address the first two objectives, our final paper (Williams and McOwan) directly explores how science could potentially help improve magic—an issue we discuss at the end of this editorial.

## Organisation

This issue showcases three papers that directly address the gap between magicians and scientists. Kuhn et al. present a psychologically based taxonomy of misdirection which directly bridges the gap between magicians' real-world knowledge of misdirection and the potential psychological mechanisms involved. The aim of this taxonomy is to organize magicians' hands-on experience and make it more accessible for people with little experience in magic. Smith et al. present a computational analysis of a conjuring trick that seeks to understand the experience of impossibility. Their approach highlights how magical effects are not simply achieved through discrete misperceptions and misattentions, but rather result from a trick's whole structure of events. Rensink and Kuhn present a framework describing how magic can further our understanding of the mind. Their framework focuses on how magic methods and effects can be used to study a range of cognitive processes. They also make the case for organizing magic tricks themselves to create a science of magic, centered around the experience of wonder that results from experiencing the impossible. On the one hand, the methods of magic provide useful tools to study cognitive processes. On the other hand, magic in itself might offer too little structure to permit a systematic exploration of its components (e.g., Lamont). Thus, whereas some of us think that studying magic is a worthwhile endeavor, others are more skeptical about this research area. The field of magic is complex, multifaceted, and certainly difficult to place under a scientific lens. It does follow some structure and overarching principles, however, and many of the challenges raised by this new science are hardly dissimilar to other burgeoning areas of psychology (Rensink and Kuhn).

This issue also features several empirical papers that use magic to study attention, memory, and reasoning. Barnhart and Goldinger present an eye-tracking study that uses a new paradigm to study misdirection and in particular the relationship between our visual experience and where we look. Similar to some previous studies, they revealed how misdirection can prevent people from seeing a fully visible event. Smith presents an eye movement study that investigated the role of audience participation on change detection, which demonstrated that participating in a task increases blindness for irrelevant features. Tompkins et al. investigated a magic trick known as the “phantom vanish,” in which assumptions can lead to erroneous perceptions of an object that was simply implied by the magician's action.

Leveraging magic to investigate cognitive mechanisms is another common theme. For example, Danek et al. focused on the mental processes involved in discovering the secrets behind magic tricks, in order to investigate insightful problem solving. Olson et al. studied how children and adults explain magic tricks differently and in particular how children provide more supernatural explanations for simple effects. The sense of wonder generated from experiencing a magic trick is central to the psychology of magic, and Danek et al. investigated the neural correlates of this unique sensation using fMRI. Another article looks at individual differences and whether all spectators are equally influenced by conjuring techniques (Wilson and French). The authors report how social influence and differences in paranormal belief govern the accuracy of reporting an ostensibly paranormal event. Finally, Mohr et al. show how experiencing an anomalous event (brought about by magic) can change cognitive markers associated with paranormal belief, in order to illustrate how magical beliefs are formed.

Becoming a professional magician requires thousands of hours of practice and most magicians learn their skills through informal social networks, Rissanen et al. interviewed prominent magicians to discover the set of skills required to become a professional and the process by which these skills are acquired. Phillips et al. explored part of this expertise in more detail by investigating how magicians are capable of deceiving their audiences through sleight of hand.

The final paper in this collection begins to examine whether science can help magicians. Williams and McOwan argue that artificial intelligence can help to improve the effectiveness of a magic trick. How science can further assist magicians create stronger effects remains one of the ultimate challenges of this nascent field.

## Future directions

Developmental psychologists harbor a long tradition of incorporating conjuring techniques into their experimental designs (e.g., Baillargeon and Devos, 1991), but in recent years, conjuring techniques have also been used to study deception in adults. For example, magic techniques have been used to secretly switch cards and induce choice blindness (Johansson et al., 2005), whilst others have used magic to convince people that a brain imaging machine could read or influence their thoughts (Olson et al., 2016). Conjuring techniques provide extremely useful experimental tools that allow us to explore psychological phenomena that would otherwise be difficult to study. We envisage that establishing firm links between magic and science will enable more researchers to use magic tricks and techniques to further enhance experimental designs.

We also envision that studying magic tricks in their own right may highlight new perspectives on cognition and likely uncover novel cognitive mechanisms (see Rensink and Kuhn; Thomas et al., 2015). This area of research is young but promising. For example, research on forcing unravels how it would be possible to tease apart decisions with and without conscious awareness (Shalom et al., 2013; Olson et al., 2015). Similarly, some classical magic effects provide intriguing insights into perceptual processes such as amodal completion (Ekroll et al., 2013), or the way in which we anticipate dynamic events (Kuhn and Land, 2006; Kuhn and Rensink, 2016). And the list goes on and on.

Many magicians remain skeptical as to whether science can promote the magical arts (e.g., Teller, 2012). This skepticism may partly result from a misunderstanding of the scientific process and perhaps because the psychology of magic is still in its early stages. Science has improved many aspects of our lives and no barriers prevent science from doing the same to magic. We sketch out at least three ways in which this trend may occur. Firstly, such a science could transfer knowledge between our current understanding of cognition and conjuring practice. For example, understanding the processing and perceptual limitations our visual system could allow magicians to exploit these bottlenecks more effectively and thus create more powerful illusions (e.g., Kuhn et al.). Secondly, scientific investigations into how and why certain tricks work will allow magicians to understand the cognitive mechanisms involved in these illusions and thus help further hone their effectiveness. For example, research on forcing (Olson et al., 2012) has revealed that people are more likely to choose certain playing cards (e.g., the Queen of Hearts) over others (e.g., the Nine of Clubs). This kind of knowledge is relevant to both magicians and behavioral scientists. As magicians and researchers continue to interact, scientists will likely uncover more practical ways to assist performers. Thirdly, we believe that the scientific method itself can help advance magic. Science is a method used to generate knowledge, and it involves asking questions that are evaluated with empirical evidence. Magicians have acquired vast amounts of knowledge about principles of deception, and they often generate this information by informally reflecting on their performances (see Rissanen et al.). This approach has lead to an impressive wealth of professional wisdom, but research in psychology has taught us that introspection can be a rather unreliable method of evaluating behavior (Nisbett and Wilson, 1977). A more objective and scientific approach to evaluating magic performance may supplement and accelerate the richness of magical information. Williams and McOwan presented a rather radical way in which artificial intelligence may help improve magic tricks—a similar approach has been used in mathematics (Diaconis and Graham, 2011)—but more subtle ways are possible too. For example, simply varying performance parameters systematically (e.g., do you choose a card physically or do you simply think of a card) combined with evaluations (e.g., post-performance questionnaires) could advance magic through systematic and rigorous explorations. Along these lines, magician Joshua Jay and scientist Dr. Lisa Grimm have recently teamed up to investigate common assumptions held by the magic community. Their research project in progress, entitled *Magic by Numbers*, is intended to provide magicians with more objective insights into how people experience magic. We trust that the continued interaction between conjurors and scientists will promote a fruitful crosstalk between psychology and the magical arts. We look forward to further realizing this joint potential.

## Author contributions

All authors listed, have made substantial, direct and intellectual contribution to the work, and approved it for publication.

### Conflict of interest statement

The authors declare that the research was conducted in the absence of any commercial or financial relationships that could be construed as a potential conflict of interest.

## References

[B1] Baillargeon R. Devos J. (1991). Object permanence in young infants - further evidence. Child Dev. 62, 1227–1246. 10.1111/j.1467-8624.1991.tb01602.x 1786712

[B2] Barnhart A. S. (2010). The exploitation of gestalt principles by magicians. Perception 39, 1286–1289. 10.1068/p6766 21125955

[B3] Beth T. Ekroll V. (2014). The curious influence of timing on the magical experience evoked by conjuring tricks involving false transfer: decay of amodal object permanence? Psychol. Res. 79, 513–522. 10.1007/s00426-014-0584-2 24941913

[B4] Binet A. (1894). Psychology of Prestidigitation. Annual Report of the Board of Regents of the Smithsonian Institution, Washington, DC: U.S. Government Printing Office.

[B5] Cavina-Pratesi C. Kuhn G. Ietswaart M. Milner A. D. (2011). The magic grasp: motor expertise in deception. PLoS ONE 6:e16568. 10.1371/journal.pone.0016568 21347416 PMC3036651

[B6] Danek A. H. Fraps T. von Müeller A. Grothe B. Oellinger M. (2014). Working Wonders? Investigating insight with magic tricks. Cognition 130, 174–185. 10.1016/j.cognition.2013.11.003 24300080

[B7] Demacheva I. Ladouceur M. Steinberg E. Pogossova G. Raz A. (2012). The applied cognitive psychology of attention: a step closer to understanding magic tricks. Appl. Cogn. Psychol. 26, 541–549. 10.1002/acp.2825

[B8] Diaconis P. Graham R. (2011). Magical Mathematics: The Mathematical Ideas that Animate Great Magic Tricks. Princeton, NJ: Princeton University Press.

[B9] Ekroll V. Sayim B. Wagemans J. (2013). Against better knowledge: the magical force of amodal volume completion. iPerception 4, 511–515. 10.1068/i0622sas 25165509 PMC4129385

[B10] Johansson P. Hall L. Sikström S. Olsson A. (2005). Failure to detect mismatches between intention and outcome in a simple decision task. Science 310, 116–119. 10.1126/science.1111709 16210542

[B11] Kuhn G. Amlani A. A. Rensink R. A. (2008). Towards a science of magic. Trends Cogn. Sci. 12, 349–354. 10.1016/j.tics.2008.05.008 18693130

[B12] Kuhn G. Land M. F. (2006). There's more to magic than meets the eye. Curr. Biol. 16, R950–R951. 10.1016/j.cub.2006.10.012 17113372

[B26] Kuhn G. Martinez L. M. (2012). Misdirection - past, present, and the future. Front. Human Neurosci. 5. 10.3389/fnhum.2011.00172 22232587 PMC3252563

[B13] Kuhn G. Rensink R. A. (2016). The Vanishing Ball Illusion: a new perspective on the perception of dynamic events. Cognition 148, 64–70. 10.1016/j.cognition.2015.12.003 26735583

[B14] Kuhn G. Tatler B. W. (2005). Magic and fixation: now you don't see it, now you do. Perception 34, 1155–1161. 10.1068/p3409bn1 16245492

[B15] Macknik S. L. King M. Randi J. Robbins A. Teller Thompson J. (2008). Attention and awareness in stage magic: turning tricks into research. Nat. Rev. Neurosci. 9, 871–879. 10.1038/nrn2473 18949833

[B16] Nisbett R. E. Wilson T. D. (1977). Telling more than we know - Verbal reports on mental processes. Psychol. Rev. 84, 231–259. 10.1037/0033-295x.84.3.231

[B17] Olson J. A. Amlani A. A. Raz A. Rensink R. A. (2015). Influencing choice without awareness. Conscious. Cogn. 37, 225–236. 10.1016/j.concog.2015.01.004 25666736

[B18] Olson J. A. Amlani A. A. Rensink R. A. (2012). Perceptual and cognitive characteristics of common playing cards. Perception 41, 268–286. 10.1068/p7175 22808582

[B19] Olson J. A. Landry M. Appourchaux K. Raz A. (2016). Simulated thought insertion: influencing the sense of agency using deception and magic. Conscious. Cogn. 43, 11–26. 10.1016/j.concog.2016.04.010 27208648

[B20] Parris B. A. Kuhn G. Mizon G. A. Benattayallah A. Hodgson T. L. (2009). Imaging the impossible: an fMRI study of impossible causal relationships in magic tricks. Neuroimage 45, 1033–1039. 10.1016/j.neuroimage.2008.12.036 19166943 PMC2680974

[B21] Shalom D. E. de Sousa Serro M. G. Giaconia M. Martinez L. M. Rieznik A. Sigman M. (2013). Choosing in Freedom or forced to choose? Introspective blindness to psychological forcing in stage-magic. PLoS ONE 8:e58254. 10.1371/journal.pone.0058254 23516455 PMC3596404

[B22] Subbotsky E. (2010). Magic and the Mind. Oxford: University Press.

[B23] Teller (2012). Teller reveals his secrets. Smithsonian Magazine.

[B24] Thomas C. C. Didierjean A. Maquestiaux F. Gygax P. (2015). Does magic offer a cryptozoology ground for psychology? Rev. Gen. Psychol. 19, 117–128. 10.1037/gpr0000041

[B25] Triplett N. (1900). The psychology of conjuring deceptions. Am. J. Psychol. 11, 439–510.

